# Evolution of the Tim17 protein family

**DOI:** 10.1186/s13062-016-0157-y

**Published:** 2016-10-19

**Authors:** Vojtěch Žárský, Pavel Doležal

**Affiliations:** Department of Parasitology, Faculty of Science, Charles University in Prague, Prumyslova 595, 252 42 Vestec, Czech Republic

**Keywords:** Protein import, Mitochondria, Evolution, Tim17, Tim22, Tim23, Oep16, Romo1, NDUFA11, TIMMDC1, Pmp24, Tmem135, Peroxisome, Plastid

## Abstract

**Background:**

The Tim17 family of proteins plays a fundamental role in the biogenesis of mitochondria. Three Tim17 family proteins, Tim17, Tim22, and Tim23, are the central components of the widely conserved multi-subunit protein translocases, TIM23 and TIM22, which mediate protein transport across and into the inner mitochondrial membrane, respectively. In addition, several Tim17 family proteins occupy the inner and outer membranes of plastids.

**Results:**

We have performed comprehensive sequence analyses on 5631 proteomes from all domains of life deposited in the Uniprot database. The analyses showed that the Tim17 family of proteins is much more diverse than previously thought and involves at least ten functionally and phylogenetically distinct groups of proteins. As previously shown, mitochondrial inner membrane accommodates prototypical Tim17, Tim22 and Tim23 and two Tim17 proteins, TIMMDC1 and NDUFA11, which participate in the assembly of complex I of the respiratory chain. In addition, we have identified Romo1/Mgr2 as Tim17 family member. The protein has been shown to control lateral release of substrates fromTIM23 complex in yeast and to participate in the production of reactive oxygen species in mammalian cells. Two peroxisomal proteins, Pmp24 and Tmem135, of so far unknown function also belong to Tim17 protein family. Additionally, a new group of Tim17 family proteins carrying a C-terminal coiled-coil domain has been identified predominantly in fungi.

**Conclusions:**

We have mapped the distribution of Tim17 family members in the eukaryotic supergroups and found that the mitochondrial Tim17, Tim22 and Tim23 proteins, as well as the peroxisomal Tim17 family proteins, were all likely to be present in the last eukaryotic common ancestor (LECA). Thus, kinetoplastid mitochondria previously identified as carrying a single Tim17protein family homologue are likely to be the outcome of a secondary reduction. The eukaryotic cell has modified mitochondrial Tim17 family proteins to mediate different functions in multiple cellular compartments including mitochondria, plastids and peroxisomes.

Concerning the origin of Tim17 protein family, our analyses do not support the affiliation of the protein family and the component of bacterial amino acid permease. Thus, it is likely that Tim17 protein family is exclusive to eukaryotes.

**Reviewers:**

The article was reviewed by Michael Gray, Martijn Huynen and Kira Makarova.

**Electronic supplementary material:**

The online version of this article (doi:10.1186/s13062-016-0157-y) contains supplementary material, which is available to authorized users.

## Background

Mitochondria use several molecular machines to deliver proteins to the correct subcompartment of the organelle [1]. All nuclear-encoded proteins destined for one of the inner mitochondrial compartments enter the intermembrane space through the Tom40 channel, then diverge onto a specialized import route to their final destination. Proteins are transported from the Tom40 channel to the mitochondrial matrix or the inner mitochondrial membrane via the inner membrane multi-subunit translocases TIM23 and TIM22 respectively [2].

Despite being very different in their overall composition both of these molecular machines are built around core proteins from Tim17 family. Members of this protein family share four transmembrane helices, which constitute the translocation channel of so far unknown molecular structure [3]. While two Tim17 family proteins, Tim23 and Tim17, constitute the channel of the TIM23 complex, the TIM22 channel is formed by only the Tim22 protein.

In addition to these mitochondrial proteins, three further Tim17 family members (Oep16, HP20 and HP30) have been identified in chloroplasts, where they participate in the plastidial protein import system [4, 5]. The identification of an amino acid sequence motif of Tim17 family proteins shared with the bacterial amino acid permease LivH led to the proposal that these bacterial, mitochondrial and plastidial proteins belong to a single family of preprotein and amino acid transporter (PRAT) proteins [6].

Moreover, following the discovery that some organisms carry a single mitochondrial Tim17 family protein [7, 8] it has been proposed that the ancestral endosymbiont possessed a single protein of the Tim17 family, from which the multiple mitochondrial paralogues were derived [9].

In this paper, we provide a comprehensive analysis of Tim17 family of proteins in eukaryotes. We identify hitherto unknown Tim17 family members in mitochondria and peroxisomes, and demonstrate the presence of multiple Tim17 family proteins in the last common ancestor of eukaryotes. Our data also suggest that proteins of the Tim17 family lack evolutionary links to the bacterial LivH-type proteins and very likely represent exclusive eukaryotic protein family.

## Results and discussion

**Fig. 1:**
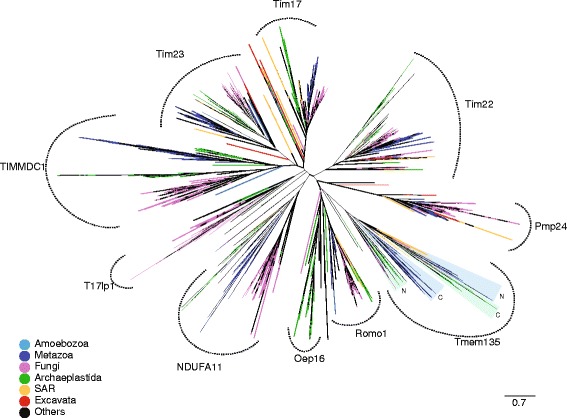
Phylogenetic reconstruction of Tim17 protein family. A tree of 5588 amino acid sequences returned by HHsearch in the Uniprot database of reference proteomes constructed using FastTree. The alignment of 136 positions was used for the reconstruction. The affiliation of protein sequences to particular supergroups of eukaryotes was color-coded. In the case of Tmem135 proteins, which contain two linked Tim17 domains, these domains were analyzed separately as N-terminal (N) and C-terminal (C) parts. *Coloured triangles* depict related N- and C-terminal Tim17 domains of Tmem135 proteins

Available genomic sequence data deposited in the Uniprot database of reference proteomes representing the major eukaryotic supergroups (Opisthokonta, Apusozoa, Amoebozoa, Excavata, Archaeplastida, SAR-Stramenopiles, Alveolata, Rhizaria) were searched by HMMs (hidden Markov models) specific to the Tim17 family (see Methods). The returned 5631 sequences (Additional file 1: Table S1) were classified by the subsequent phylogenetic analysis (Additional file 1: Table S1, Additional file 2: Table S3). The analysis uncovered the presence of prototypical sequence groups specific to Tim17, Tim22 and Tim23, as well as additional related groups (Fig. 1).

Inspection of Tim22 subgroup showed that in addition to the mitochondrial proteins, two plastidial Tim17 family proteins, HP20 and HP30, represent Tim22 orthologues [5] (Additional file 1: Table S1). Analogously to mitochondrial Tim22, both HP20 and HP30, take part in the import of the inner membrane plastidial proteins [5]. The relationship is further supported by the presence of one or both invariant cysteine residues (Additional file 3: Figure S1). In Tim22, the residues were shown to participate during the Mia40- dependent protein import into mitochondria [10] and/or contribute to the TIM22 complex stability via the formation of an intramolecular disulphide bond [11]. The lack of MIA pathway in the plastids suggests that plastidial Tim17 proteins do not use disulfide relay system during the import and the cysteine residues may, at least in case of HP20, also participate in the stabilization of the translocase complex in the plastid.

### Additional members of the Tim17 family

**Fig. 2:**
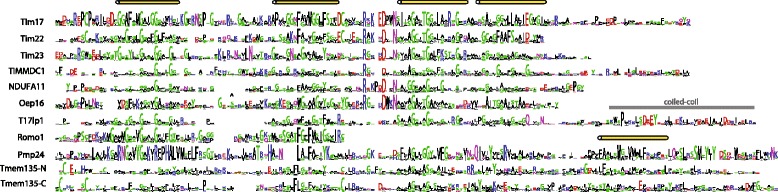
Comparison of different groups of Tim17 family proteins. Aligned HMM logos of particular groups of Tim17 family proteins show the overall similarity and diversity of the protein family. The* yellow cylinders* depict the hydrophobic transmembrane regions, while the *gray bar* highlight the coiled-coil domain in T17lp1 proteins. The following reference sequences can be used for particular logos: Tim17 - Q99595 (residues 1–171), Tim22 - Q9Y584 (residues 61–194), Tim23-O14925 (residues 64–209), TIMMDC1 - Q9NPL8 (residues 61–271), NDUFA11 - Q86Y39 (residues 6–141), Oep16 - Q9ZV24 (residues 17–148), T17lp1- U9W558 (residues 41–226), Romo1 - P60602 (residues 7–79), Pmp24 - Q9Y6I8 (residues 17–207) and Tmp135 Q86UB9 - N- part (residues 10–200) and C- part - (residues 242–433)

**Fig. 3:**
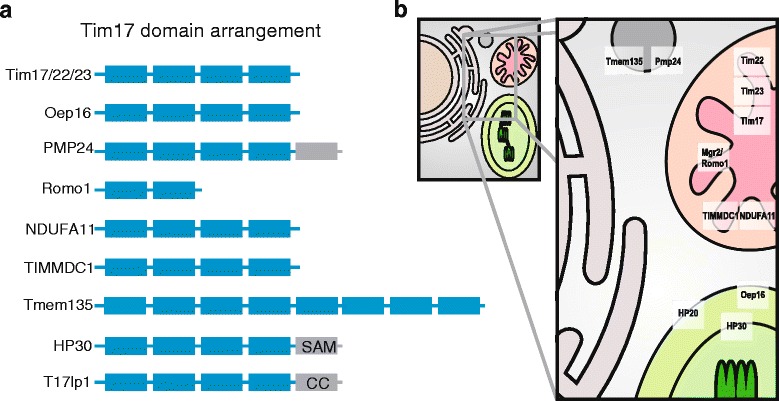
Characteristics and cellular distribution of Tim17 protein family members. **a** The Tim17 family proteins were analysed for the presence of the transmembrane domains (*gray rectangles*) and additional protein domains (*blue rectangles*) as described in Methods. SAM-sterile alpha motif, CC-coiled coil domain. **b **Mitochondrial inner membranes contain Tim17, Tim22, Tim23 and Mgr2, which participate in the import and the membrane assembly of the mitochondrial proteins. Human orthologue of Mgr2, Romo1 has been linked to production of reactive oxygen species. TIMMDC1 and NDUFA11/Nuo21.3 /B14.7 assist in the assembly of mitochondrial complex I. Plastidial HP30 and HP20 participate in import of inner membrane proteins via the inner and the outer membranes, respectively. Oep16 is the outer membrane channel for amino acids, which also contributes to plastidial protein import. The function of peroxisomal proteins PMP24 and Tmem135/PMP52 remains unknown

Our HMM-based searches identified several additional Tim17 family members (Fig. 1, Additional file 4: Table S2). These proteins include mitochondrial and also peroxisomal proteins with diverse but always membrane associated functions (Fig. 3b). In general, the domain structure of the protein family members is derived from the basic architecture of four membrane spanning helices rich in glycine residues, often arranged as glycine zipper motives (Figs. 2 and 3a). However, several family members carry different number of TMDs, which can thus range between two to eight TMD segments (Figs. 2 and 3a). Other common features include conserved positively and negatively charged residues between the second and the third TMDs, although the conservation is less obvious among peroxisomal proteins.

In mitochondria, we found a distinct group of Tim17 family sequences, which includes human NDUFA11, fungal Nuo21.3 and plant B14.7. These proteins have previously been shown to be a component of mitochondrial complex I, participating in the complex assembly and connecting the membrane and peripheral arms of the complex. In addition to complex I, the plant protein B14.7 also participates within the TIM23 complex together with Tim23 and Tim17 [12].

Similarly, TIMMDC1 has recently been found to function as an assembly factor for the human mitochondrial complex I [13, 14] and the affinity of TIMMDC1 and NDUFA11, to Tim17 protein family was already shown [13, 14]. While both groups of proteins carry four TMDs spanning the inner mitochondrial membrane, as typical for Tim17/Tim22/Tim23 proteins (Fig. 3a), our analysis showed that TIMMDC1 is phylogenetically distinct from the NDUFA11/Nuo21.3 /B14.7 subgroup (Fig. 1). Together, the participation of Tim17 family proteins in the complex I assembly provide another link between the mitochondrial protein import and the respiratory chain [15].

**Fig. 4:**
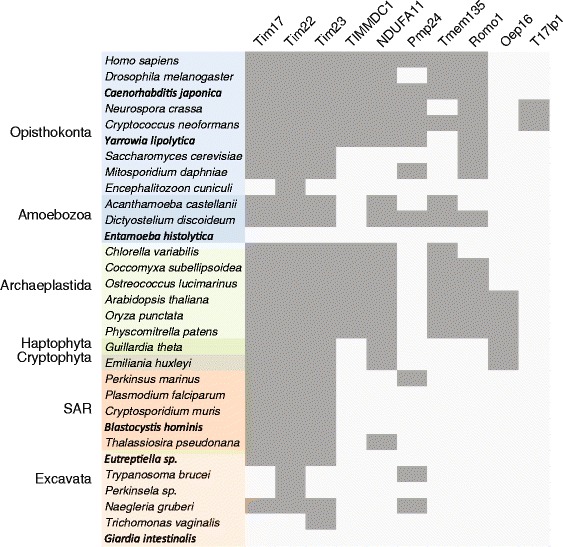
Distribution of Tim17 family proteins in eukaryotes. The presence of particular Tim17 proteins in selected eukaryotic genomes representing supergroups of eukaryotes. *Gray* or *white* fields depict the respective presence or absence of the components

Interestingly, our analyses found homology to the Tim17 family in the yeast Mgr2 [16] and its human ortholog Romo1 [17]. These proteins carry only the first two TMDs of the Tim17 protein family (Figs. 2 and 3a, Additional file 5: Figure S2). Recent reports on the role of Mgr2 and Romo1 suggest different roles for them in mitochondrial biology. Mgr2 was found to be a component of the yeast TIM23 complex, where it controls the lateral release of the membrane proteins from the translocation channel [18]. The TIM23 translocase is thus built of three specialized Tim17 family proteins: a pore forming Tim23 [19], Tim17 maintaining the translocase twin pore architecture [20] and a gating Mgr2 [18]. Romo1, however, has been reported to take part in the mitochondrial ROS generation pathway leading to apoptosis [21], and to affect the mitochondrial morphology [22]. Whether these reported functional differences between Mgr2 and Romo1 reflect lineage-specific roles or rather point to their common function is yet to be demonstrated. In any case, the presence of Mgr2/Romo1 orthologs in diverse groups of eukaryotes, Opisthokonta, Amoebozoa, Archaeplastida and SAR, suggests its conserved general role in mitochondrial biology (Fig. 4).

Two of the proteins identified in our analysis, PMP24 and Tmem135 were previously shown to localise to peroxisomes. In agreement with the Pfam database, which has assigned PMP24 into the Tim17 protein family [23], this is thus the first report on the presence of Tim17 homologues in this organelle. PMP24 was originally isolated from rat liver peroxisomes [24] as an integral membrane protein [25]. Despite being highly conserved and present across eukaryotic lineages except Archaeplastida (Fig. 4), its function remains entirely unknown. The protein consists of four transmembrane domains homologous to other Tim17 proteins with additional C-terminal transmembrane domain, that is specific only to the peroxisomal orthologues (Figs. 2 and 3a). Tmem135, a 52 kDa protein, also referred to as PMP52, was originally identified by mass spectrometry of purified peroxisomes [26, 27]. Based on the expression profiling the protein was suggested to take a part in the fatty acid metabolism [28, 29]. Tmem135/PMP52 is predicted to carry eight TMDs, which correspond to two Tim17 protein family domains. The protein is present in all eukaryotic supergroups, however the ambiguous relationship between the N- and C-terminal Tim17 domains indicate that these have been swapped during evolution (Fig. 1). That a member of the originally described mitochondrial protein family was found in the peroxisomes has a precedent in the case of the peroxisomal ADP/ATP carrier PMP34, a member of mitochondrial carrier protein family [30]. Considering that a Tim17 protein (Tim22) assembles the mitochondrial carriers into the inner mitochondrial membrane, it is tempting to speculate that PMP24 mediates the insertion and the assembly of PMP34 into the peroxisomal membrane.

In addition, a new group of so far uncharacterized Tim17-like proteins has been uncovered in our analyses, referred to as Tim17-like proten 1 (T17lp1) (Fig. 1). The members of the group can be found predominantly in fungi including the model organism *Neurospora crassa* (U9W558_NEUCR) and also stramenopiles, green and red algae (Additional file 1: Table S1 and Additional file 4: Table S2). According to the predictions the proteins possess four membrane spanning regions and a C-terminal coiled-coil domain (Figs. 2 and 3a).

Finally, the inspection of current pfam alignments showed that except Romo1/Mgr2 proteins, which belong to PF10247, additional Tim17 members except the newly defined T17lp1 proteins are already included in Tim17 family PF02466.

### Evolutionary distribution of Tim17 family proteins

**Fig. 5:**
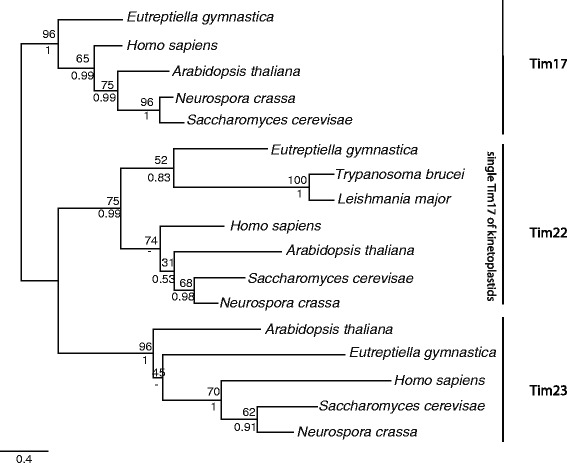
Phylogenetic reconstruction of Tim17 family proteins from *Eutreptiella gymnastica*. Tim17 family amino acid sequences from euglenid *E. gymnastica* and kinetoplastids *Trypanosoma brucei* and *Leishmania major* were aligned with representative Tim17/Tim22/Tim23 protein sequences, and phylogeny was constructed by Phyml (*lower values*) and MrBayes (*upper values*)

The genomes of *Trypanosoma brucei* and other related kinetoplastids have been shown to contain only a single mitochondrial Tim17 protein [7, 8]. It has been hypothesized that the presence of a single mitochondrial Tim17 family protein could correspond to the ancestral design of the translocase before its diversification into multiple specialized proteins [9]. However, our analysis here identified three Tim17 family protein sequences in the euglenid *Eutreptiella gymnastica.* We employed two independent means of phylogenetic analysis and in each case found that these sequences correspond to the three prototypical Tim17, Tim22 and Tim23 proteins (Fig. 5). This finding strongly supports the presence of these three specialized Tim17 family proteins in the mitochondria of the ancestor of all Euglenozoa, which include *T. brucei*.

Our HHsearch analysis supports the presence of just a single Tim17 family protein in kinetoplastids reported earlier [7, 8] but this likely represents a secondary simplification of the mitochondrial protein translocation machines. There is a precedent for this type of simplification; an even more striking secondary reduction of the mitochondrial protein import apparatus has previously been observed in eukaryotes inhabiting anaerobic environments, exemplified by the loss of all detectable Tim17 family proteins from the mitochondria-related organelles of *Giardia intestinalis* and *Entamoeba* species [31, 32].

We have shown that every eukaryotic supergroup includes species, which encode all three of the prototypical mitochondrial Tim17, Tim22 and Tim23 proteins (Fig. 4). It is thus highly likely that the last eukaryotic common ancestor (LECA) contained this triad in its inner mitochondrial membrane. According to our analyses, it is also likely that peroxisomes of the LECA accommodated a Tim17 family protein in their membranes. Provided that, in contrast to the peroxisomal Tim17 family protein(s), all three mitochondrial members represent the essential genes in the organisms studied, mitochondrion is very likely the original place of action of Tim17 protein family.

Does this imply that Tim17 homologue was present in the membranes of the bacterial progenitor of mitochondria? Previously, the similarity between Tim17 family proteins and a membrane component of the bacterial inner membrane transport system for the branched-chain amino acids, LivH [33], led to the proposal of so-called PRAT family (the preprotein and amino acid transporter) [6]. In order to test the bacterial link to the eukaryotic Tim17 family proteins we searched all available bacterial protein sequences deposited in the UniProt Knowledgebase with Tim17 specific HMMs built from our complete dataset of all identified Tim17 homologues or Pfam seed alignment. The returned bacterial proteins contained a glycine-zipper motif (GxxxGxxxG) (Pfam clan CL0500) and interestingly, the highest ranking hits were from the alphaproteobacterial group of Rhizobiales. However, except to the glycine- zipper motif, these proteins did not share other Tim17 characteristics. In support of that, when the glycine-zipper motif was masked, the similarity score dropped dramatically (data not shown).

Similarly, searching the prokaryotic databases with PRAT family motif (G/A)X_2_(F/Y)X_10_RX_3_DX6(G/A/S)GX_3_G number of protein sequences returned with no obvious link to Tim17 family of proteins (Additional file 6: Table S4). We thus conclude that there are similar domains in bacterial proteins, however, these cannot be used to unambiguously pinpoint the prokaryotic ancestor of the Tim17-like protein family.

Thus, we propose that Tim17 proteins represent unique eukaryotic protein family, which has first enabled the mitochondrion to import its proteins, while later provided additional roles in the peroxisome biology, plastidial protein import, the assembly of the mitochondrial complex I and the mitochondrial apoptotic pathway.

## Methods

### Tim17 homologues detection and classification

In order to conduct an exhausting search of Tim17-like homologs, we employed an iterative search of the Uniprot database of reference proteomes which contains 5631 proteomes from all domains of life. Manually selected Tim17 homologs from the Swissprot database were used as query for the search. In each iteration, the discovered homologs were divided into groups by a 40 % identity cutoff using CD-HIT. Each group was then aligned and searched against the Uniprot database of reference proteomes using Hmmer [34]. Hits were selected using a very shallow cutoff (e-value < = 1) and checked for homology against the query using Hhsearch. Hits with probability above 90 % were used as a query for next iteration. After 10 iterations we collected 6246 possible Tim17 homologs. These were then thoroughly checked using Hhpred procedure – for each sequence a HMM profile was created using PSI-BLAST and compared to the alignment of unequivocal Tim17 homologs where the glycine-zipper positions were masked to avoid unspecific hits of non-homologous regions with glycine-zippers. During the process homologous sequences were attached to the original alignment. We used the resulting alignment for the inference of phylogeny using FastTree [35].

### Characterization of Tim17 family members

The amino acid sequences of the identified proteins were analysed by BLAST and Hhpred in order to detect possible homology to other protein families. The occurrence of the transmembrane domains was predicted using Phobius [36], TMHMM [37] and TMpred (http://www.ch.embnet.org/software/TMPRED_form.html).

### Tim17 homologues in Euglenozoa

The MMETSP database of transcriptomic data was searched using Hmmer [34] and homologues of Tim17, Tim22 and Tim23 were recovered in the sequence data of *Eutreptiella gymnastica*. A dataset of known homologues of Tim17, Tim22 and Tim23 (*Homo sapiens*, *Neurospora crassa*, *Saccharomyces cerevisiae*, *Arabidopsis thaliana*), mitochondrial Tim17-like proteins of kinetoplastids (*Trypanosoma brucei*, *Crithidia fasciculata*, *Leishmania major* and *Bodo saltans*) and *E. gymnastica* sequences was aligned using Mafft (--maxiterate 1000 --localpair options) [38]. The alignment was trimmed using BMGE (matrix BLOSUM30 and block size 1) [39]. A phylogeny was constructed using Phyml [40] and MrBayes [41] with the WAG substitution model.

### PRAT motif in prokaryotes

The prokaryotic protein sequences were searched by a PRAT motif [GA]-x-x-[FY]-x-x-x-x-x-x-x-x-x-x-R-x-x-x-D-x-x-x-x-x-x-[GAS]-G-x-x-x-G using Motif search at Kyoto University Bioinformatics Centre (http://www.genome.jp/).

## Conclusions

This study reports on the broad analyses of the Tim17 family of proteins in eukaryotes. In addition to the prototypical family members of the mitochondrial protein import machines, the family includes assembly factors for the mitochondrial complex I and plastid protein import components. We show that additional members of the protein family function in mitochondria and peroxisomes and that the LECA was already equipped with multiple Tim17 family proteins in its mitochondria and peroxisome. Moreover, currently there is no indication that the Tim17 protein family was present in the bacterial ancestor of mitochondria.

## Reviewers’ comments

### Comments and responses to the original submission

#### Reviewer’s report 1: Michael Gray, Dalhousie University, Canada

“This manuscript describes the results of a straightforward data-mining analysis to explore the functional and phylogenetic diversity of the Tim17 protein family. The authors demonstrate that all eukaryotic supergroups contain the three core proteins of the TIM22 and TIM23 protein translocases of the inner mitochondrial membrane, i.e., Tim17, Tim22 and Tim23. They conclude that the last eukaryotic common ancestor (LECA) had all three proteins, arguing that the single Tim17 family member in kinetoplastid protozoa represents a simplification, rather than the ancestral state. The authors further identify Tim17 family members involved in different functions and located in different subcellular organelles (peroxisome as well as mitochondrion). Finally, the authors fail to find any convincing evidence indicating an evolutionary connection between the Tim17 family and bacterial LivH (amino acid permease), concluding that the Tim17 family is exclusive to eukaryotes.

This paper is an interesting and generally well-executed contribution to our understanding of the evolution of the mitochondrial proteome. The authors have compiled a valuable data set and used it to make novel phylogenetic and functional inferences about the Tim17 protein family. This study nicely illustrates the utility of comprehensive comparative analyses in robustly supporting evolutionary conclusions.”

Reviewer recommendations to authors

“I have some reservations about how effectively the automated procedure used by the authors has resulted in a truly comprehensive retrieval of relevant sequences. For instance:

• Inspection of Fig. 3 for Amoebozoa (a eukaryotic supergroup with which I am particularly familiar) indicates absence of Tim23 from Acanthamoeba castellanii and Dictyostelium pupureum but its presence in both D. discoideum and D. fasciculatum. In fact, Tim23 was previously identified in a proteomic analysis of A. castellanii mitochondria [Gawryluk et al. (2014) J. Proteomics 109, 400–416; XP 004336406.1]. The corresponding D. purpureum protein is XP_003287004.1.

Author’s response: *Thanks. Unfortunately, this was caused by incomplete set of analyzed sequences, which we collected. We have re-done the analyses using the whole Uniprot database. In order to avoid false negatives we thus analyzed the whole Uniprot database of the 5631 reference proteomes from all domains of life. Doing so we have largely enriched the classification of Tim17 family members and their distribution in different groups of eukaryotes. This time the returned sequences contain the missed hits as noticed by the reviewer.*

• Figure 3 shows A. castellanii lacking NDUFA11, yet the corresponding sequence is included in Additional file 1: Table S1. NDUFA11 is shown as largely missing from most organisms in Fig. 3, yet Cardol (2011) [Biochim. Biophys. Acta 1807, 1390–1397] has demonstrated that it is widely distributed in all eukaryotic supergroups.

Author’s response: *In this case we wrongly labeled the absence of NDUFA11 in Fig.* *4* *. The overall analysis was re-done and this mistake was corrected.*

• A. castellanii Trem125 was identified and annotated as an “uncharacterized conserved protein” (N375) in the study of Gawryluk et al. (2014) cited above. The complete Trem125 sequence (as well as a corrected sequence for A. castellanii Tim22) can be found in Gawryluk et al. (2014) Data in Brief 1, 12–14.

Author’s response: *Thanks. We have corrected that.*

• Romo1 and Oep16 are indicated as being present in A. castellanii in Fig. 3, but the corresponding sequences are not listed in Additional file 1: Table S1.

Admittedly, these omissions/inconsistencies do not affect the authors’ overall functional and evolutionary conclusions. However, some judicious manual searches to fill in gaps coupled with reference to previously published mitochondrial proteome analyses, as well as careful checking for consistency between Fig. 3 and Additional file 1: Table S1, would make the latter a more complete, accurate and useful data set for other workers in the field.”

Author’s response: *Thanks. We have corrected these inconsistencies. Moreover, by using only Uniprot database, all sequences can now be more easily identified. Also, the taxonomic classification has been done to better detail.*

Minor issues

“line 138: “indentified” should be “identified”

Author’s response: *done*

Figure 1: “N” and “C” should be explained in the legend.”

Author’s response: *done*

#### Reviewer’s report 2: Martijn Huynen, Radboud University, Netherlands

Reviewer summary

“The manuscript handles an interesting subject, but I do find rather superficial in its analyses, in its comparisons with the literature (most of what it mentions in the abstract as new, is known) and in its discussion of the results. Often the logic the authors suggest in their language does not make sense to this referee. I do suggest the authors go back to the “drawing board”, do there analysis more thoroughly and spend some time on interpreting their results.”

Reviewer recommendations to authors

1) “There is no explanation of the choice of species, some model species (e.g. a model species of the fungi for mitochondrial research like Y. lipolytica are missing) while three species of the Dictyostelium genus are present. Are these genomes particularly well sequences/annotated?

There should be reciprocal checking of homologs, specifically for the “borderline” cases. In general E-values are used find homologs, not a “probability cutoff”.

Author’s response: *Thanks. Our intention was to cover all major eukaryotic supergroups. However, after the review we decided to run more thorough analyses on the whole Uniprot database of the reference 5631proteomes instead of selected proteomes. The detailed results of the analyses are submitted as Additional files, some of which are shown as Fig.* *4* *of the main text. We have also performed manual checks for the borderline sequences to eliminate false positives*.

2) At least one reference is incomplete (Mokranjac and Neupert).

Author’s response: *The reference was corected*

3) The paper could have done with a more thorough analysis of the literature. The homology between NDUFA11, TIMMDC1 and the TIM17 family was already known in 2013. The authors actually cite a paper where that is mentioned but somehow forgot to read it? (Andrews et al., PNAS 2013). There are actually multiple papers mentioning this homology. Similarly PMP24 has already been part of this family in PFAM, and this should be mentioned.

Author’s response: *Our mistake not referring to the previously published homology between NDUFA11 and TIMMDC1 and Tim17 is unfortunate. We have corrected that and also toned down the title of the section of the manuscript accordingly. Reference to Pfam database was also included.*

4) What does appear new, is that they find ROMO1/MGR2 to be homologous to the TIM17 family. Given the novelty of that, and given that MGR2 is part of a complex with the TIM17 homolog TIM23, and given the recent interest in its function, this does deserve more attention, including an alignment and interpretation of conserved residues in light of what is known about these proteins (their interactions in the membrane).

Author’s response: *We have added the alignment of Romo1/Mgr2, Tim17 and Tim23 sequences as Additional file* *5* *: Figure S2. However, so far there is limited data available on the role of particular amino acid residues of Romo1/Mgr2 or its interaction with Tim23 or Tim17.*

*In the repeated analyses using much larger dataset we have also uncovered additional group of proteins carrying a C-terminal coiled coil domain, referred to as T17lp1 (Tim17-like protein 1).*

5) The lack of homology between LivH and TIM17 has been noted before, even in this journal (Jeferson Gross and Debashish Bhattacharya Biology Direct 2012, which happens to also have been reviewed by this referee).

Author’s response: *Yes, we and others have been aware of the lack of homology between LivH and Tim17, which was originally described by Rassow et al., 1999. However, the preposition made against the two proteins relationship were usually based on unsuccessful pair-wise sequence analyses such as blast (e.g. the article by Gross and Bhattacharya, 2012). Therefore, we have felt that more sensitive analysis had to done. We have added following paragraph to the text: “*[6]. In order to test the bacterial link to the eukaryotic Tim17 family proteins we searched all available bacterial protein sequences deposited in the UniProt Knowledgebase with Tim17 specific HMMs built from our complete dataset of all identified Tim17 homologues or Pfam seed alignment. The returned bacterial proteins contained a glycine-zipper motif (GxxxGxxxG) (Pfam clan CL0500) and interestingly, the highest ranking hits were from the alphaproteobacterial group of Rhizobiales. However, except the the glycine- zipper motif, these proteins did not share other Tim17 characteristics. In support of that, when the glycine-zipper motif was masked, the similarity score dropped dramatically (data not shown).”

6) The table with the phylogenetic distribution of the protein subfamilies appears inconsistent with the results: e.g. the authors say that they find a NDUFA11 protein in plants, and it appears in their phylogeny, but then do not show an NDUFA11 (which appears under many names in the manuscript & supplement) ortholog in the table. In general I do find the many “holes” in the table suspiscious. I suggest they do a better job of their homology searches, and also search against the DNA/RNA sequences of the species they are analyzing to search for genes that were not predicted as such.

Author’s response: *In order to minimize the false negatives in our analyses, we have completely re-done the analyses using the entire Uniprot database of reference proteomes as a sequence source. However, still some “holes” remain. The detailed table of Tim17 proteins can be found in Additional file* *1* *: Table S1 and Additional file* *4* *: Table S2.*

7) In Fig. 1 the “Oep16” clade is not well defined. One cannot include het two yellow branches and one green branch in Oep16 based on this tree. Please clarify.

Author’s response: *The entire Fig.* *1* *has been changed on the base of the different and larger dataset.*

8) The following two sentences do not make much sense:

“Analogously to mitochondrial Tim22, both HP20 and HP30, participate in the import of the inner membrane plastidial proteins. The presence of the conserved cysteine residues suggests that these also participate in the stabilization of the translocase complex in the plastid as the redox-regulated protein import analogous to MIA pathway does not occur here.”

Why does the absence of the MIA complex support that the cysteines are involved in stabilization?

Author’s response: *Given that the cysteine residues in Tim22 have been show to serve for two purposes – the complex stabilization and/or the Mia40 dependent import, we propose that, when the MIA pathway is missing, the function of the cysteine residues is rather to stabilize the mature protein complex.*

9) A sentence like “The peroxisomal localization of Tim17 family protein is reminiscent to the peroxisomal ADP/ATP carrier PMP34, a member of mitochondrial carrier protein family (Wylin et al. 1998).” also carries more meaning than it actually has.

Author’s response: *Yes, the expression is not precise and it was rephrased to the following:*

*“That a member of the originally described mitochondrial protein family was found in the peroxisomes has a precedent in the case of the peroxisomal ADP/ATP carrier PMP34, a member of mitochondrial carrier protein family [30]. Considering that a Tim17 protein (Tim22) assembles the mitochondrial carriers into the inner mitochondrial membrane, it is tempting to speculate that PMP24 mediates the insertion and the assembly of PMP34 into the peroxisomal membrane.”*

10) “This fact is accentuated by the presence of one or both invariant cysteine residues (Additional file 3: Figure S1), which are required for the Mia40- dependent import of Tim22 into mitochondria (Wrobel et al. 2013) and contribute to TIM22 complex stability via the formation of an intramolecular disulphide bond.” How can one cysteine create an intramolecular disulphide bond?

Author’s response: *Of course, the formation of the intramolecular dissulfide bond was meant for the situation when both cysteine residues are present - the whole paragraph was rephrased as follows:*

*“Inspection of Tim22 subgroup revealed that in addition to the mitochondrial proteins, two plastidial Tim17 family proteins, HP20 and HP30, represent Tim22 orthologues [5] (Additional file* *1* *: Table S1). Analogously to mitochondrial Tim22, both HP20 and HP30, take part in the import of the inner membrane plastidial proteins [5]. The relationship is further supported by the presence of one or both invariant cysteine residues (Additional file* *3* *: Figure S1). In Tim22, the residues were shown to participate during the Mia40- dependent protein import into mitochondria [10] and/or contribute to the TIM22 complex stability via the formation of an intramolecular disulphide bond [11]. The lack of MIA pathway in the plastids suggests that plastidial Tim17 proteins do not use disulfide relay system during the import and the cysteine residues may, at least in case of HP20, also participate in the stabilization of the translocase complex in the plastid”.*

11) I do find Fig. 2 not enough evidence that ROMO1 is homologous to the first two transmembrane regions from TIM17.”

Author’s response: *Now the support for this claim can be found in the Additional file* *5* *: Figure S2, reference to it was added to the text.*

#### Reviewer’s report 3: Kira Makarova, NLM, NIH, USA

“The manuscript describes a relatively straightforward but accurate sequence and comparative genomic analysis of Tim17 family. The significance, originality and novelty of presented results are not very high, but the analysis is done carefully on a large genome set and the paper still could be useful for those who are interested in evolution of the components of mitochondrial translocases.”

Reviewer recommendations to authors

“The manuscript describes a relatively straightforward but accurate sequence and comparative genomic analysis of Tim17 family. These proteins were first characterized as components of protein translocase of inner mitochondrial membrane and are essential for mitochondria functioning. As it is the case for many membrane protein families, they are very poorly annotated and this analysis could be helpful to improve annotation of these important proteins. In addition the family members are found in perxisomes and plastids. I don’t have much criticism for the methods applied and I find that conclusions are generally justified, pending some answers to the questions below. I however have a number of suggestions and questions to the authors.

1. The title of the paper is misleading since all the different functions of the superfamily members have been characterized before elsewhere. As far as I can see there is no additional groups found in this paper which function has not been known before. This paper simply presents comparative genomic analysis of the family but does not analyze their diverse roles. Thus the title should better reflect the work which has been done here.

Author’s response: *Yes, we agree and we have change the title to better described the content of the paper to:*

*“Evolution of the Tim17 protein family”*

2. Furthermore I suggest that authors should rephrase the abstract considerably, since as mentioned above they have not studied function of these proteins. Also the novelty of the presented results is not obvious from the abstract. It should be clarified if authors detected any new subfamilies or showed similarity between the families that was not detected before or showed relationships between subfamilies that have not been shown before. See also the next suggestion.

Author’s response: *Yes, the abstract has been modified accordingly*

3. As far as I can tell there are two pfam profiles that include Tim17 family members (Tim17 Tim17/Tim22/Tim23/Pmp24 family) and pfam10247 (Romo1). It should be clearly stated if authors have identified any subfamilies that are not present in the respective pfam alignments. Note that the protein sequence (such as NDUFA11) could be included in the pfam alignments but have not identified by this ID. Authors should check that. This is also important to understand if additional pfam alignments should be made to cover the whole family better or if the ones that exist are sufficient.

Author’s response: *Our inspection of the pfam alignments confirmed that NDUFA11, TIMMDC1, Oep16 sequences except the newly define T17lp1 proteins are already included in the dataset of Tim17 Tim17/Tim22/Tim23/Pmp24 family - we have included that information in the text.*

4. Alignment of a few representatives of each subfamily should be shown as a main figure and it should be discussed. In particular if still there is a motif/consensus characteristic for the whole family (discuss if the new motif (if any) is different from the old one called PRAT family motif), which region of the sequences is the most conserved, etc.

Author’s response: *The aligned HMM logos of different Tim17 family members were added to the paper as Fig.* *2*.

5. Authors made a considerable effort trying to provide some supporting evidence for the previously published hypothesis that Tim17 family originated from LivH amino acid transporter component. That hypothesis was very weak so not surprising that they could not find any support for it. But the conclusion that Tim17 family is specific to eukaryotes is also dangerous, since membrane proteins evolve fast and it is difficult to identify there distant homologs. Authors mentioned that HHpred search identifies a number of bacterial families with G zipper motif (albeit with low confidence). I would recommend authors to analyze this similarity very carefully with respect of additional potential shared motifs, number of transmembrane domains, relevance to electron transport complexes or any other relevant sequence features. The paper would be much more interesting if authors could present a better hypothesis on the origin of Tim17 family.

Author’s response: *During our searches we found number of hits among bacterial proteins that contain a glycine-zipper motif (Pfam clan CL0500), interestingly the highest ranking hits were from the alphaproteobacterial group Rhizobiales. In addition to the glycine- zipper motif these proteins, however, they did not share other Tim17 characteristics. In support of that, when the glycine-zipper motif was masked, the score dropped dramatically. We thus conclude that there are similar domains in bacterial proteins, these can not be used to unambiguously pinpoint the prokaryotic ancestor of the Tim17-like protein family. We have added this information to the manuscript.*

6. Are there any similarity (sequence, organization of subunits, number of transmembrane domains, origin, phyletic distribution) of the Tom40 channel and Tim17 based complexes? It would be interesting to read some discussion to this extent. Also in the HHpred searches there is a weak similarity to Mitochondrial uncoupling protein 2 (PDB: 2lck) and Tom5. Could any of those be real based on additional considerations mentioned above?

Author’s response: *So far, there has been no similarity found in any aspects of the TIM and TOM complexes and our analyses support that. Tom40 is a beta-barrel protein, which can be found only in the outer membranes of gram-negative bacteria, plastids and mitochondria. Also the receptor subunits and small Toms are exclusive to the TOM complex. The relationship of Tim17 protein to mitochondrial uncoupling protein 2 (and other members of the mitochondrial carrier protein family) rather reflects a similarity among the transmembrane segments of different mitochondrial inner membrane proteins than the true affiliation of the proteins.*

1. Mention (in the Fig. 1 legend or methods) how many position of alignment were used for tree reconstruction and if positions with many gaps alignment were filtered. Also please provide a complete tree in Newick format as a supplement.

Author’s response: *The information and Newick format of the tree was added as a supplement (Additional file* *2* *: Table S3).*

2. Figure 5 is nice but not needed (everything shown in the figure is clearly stated in the text) or at least it can be merged to the Fig. 2, which is also dispensable but more useful.

Author’s response: *Yes, we agree, figure was meant as a graphical summary of the Tim17 evolution. We have merged Fig.* *5* *to Fig.* *2*.

3. Additional file 1: Table S1 does not indicate the database of origin of the respective sequence and the IDs look messy. It would be good to correct that.”

Author’s response: *We have used only Uniprot IDs in the revision.*

### Comments and responses to the revision

#### Reviewer’s report 2: Martijn Huynen, Radboud University, Netherlands

1) I do not have much to add to my previous comments. Small remarks: “The lack of MIA pathway in the plastids suggests that the cysteine residues may, at least in case of HP20, also participate in the stabilization of the translocase complex in the plastid.” How does the lack of the MIA pathway support this? Please clarify this in the manuscript (not just in the answer to me). The MIA dependent import pathways works via the oxidation of the cysteines. In its absence you actually have the problem that it is not clear how these cysteines are oxidized.

Author’s response: *We have rewritten the paragraph to the following:” The lack of MIA pathway in the plastids suggests that plastidial Tim17 proteins do not use disulfide relay system during the import and the cysteine residues may, at least in case of HP20, also participate in the stabilization of the translocase complex in the plastid.*

2) Regarding the lack of homology between LivH and TIM17. Please be so kind to cite the previous discussion about this, as I have asked before and show what you did differently.

Author’s response: *Concerning the homology between LivH and TIM17. We have added following paragraph to the text: “[6]. In order to test the bacterial link to the eukaryotic Tim17 family proteins we searched all available bacterial protein sequences deposited in the UniProt Knowledgebase with Tim17 specific HMMs built from our complete dataset of all identified Tim17 homologues or Pfam seed alignment. The returned bacterial proteins contained a glycine-zipper motif (GxxxGxxxG) (Pfam clan CL0500) and interestingly, the highest ranking hits were from the alphaproteobacterial group of Rhizobiales. However, except the the glycine- zipper motif, these proteins did not share other Tim17 characteristics. In support of that, when the glycine-zipper motif was masked, the similarity score dropped dramatically (data not shown).”*

3) “Inspection of Tim22 subgroup revealed that in addition to the mitochondrial proteins, two plastidial Tim17 family proteins, HP20 and HP30, represent Tim22 orthologues [5] (Additional file 1: Table S1).” How can HP20 and HP30 be Tim22 orthologues when A. thaliana also has actual Tim22 orthologs? (At1g18320 and At3g10110).

Author’s response: *In our bioinformatic analyses HP20 and HP30 cluster together with Tim22 proteins. With respect to the conserved function of both proteins in the protein transport, we have called them as Tim22 orthologues (and not paralogues).*

4) The affinity of TIMMDC1 and NDUFA11, to Tim17 protein family was already suggested“ This was more than just ’suggested” in that paper. They showed an actual alignment and mentioned the PFAM family.

Author’s response: *We have corrected that.*

#### Reviewer’s report 3: Kira Makarova, NLM, NIH, USA

The manuscript describes a relatively straightforward but fairly accurate sequence and comparative genomic analysis of Tim17 family. These proteins are components of protein translocase of inner mitochondrial membrane and are essential for mitochondria functioning. As it is the case for many membrane protein families, they are very poorly annotated and this analysis could be helpful to improve annotation of these important proteins in eukaryotic genomes. I don’t have a lot of criticism for the methodology and I find that conclusions are generally justified. However authors’ answers to a few questions below could clarify some additional details regarding results and conclusions mentioned in the main text.

1) Abstract still needs another round of refinement and restructuring. Results and novelty should be better clarified. “Two further Tim17 family proteins are present in the peroxisomal membranes” – this is not a result of this work. Inference about LECA should go to conclusions.

Author’s response: *We have changed the abstract accordingly.*

2) Could authors provide here more technical details of their attempts to identify homologs in prokaryotes (HHpred probabilities, compatible or incompatible number of TM domain, etc.)?

Author’s response: *We have added the information into the manuscript.*

3) Any speculation on the origin of Tim17 membrane proteins? It would be interesting to read more discussion on the comparison (sequence, organization of subunits, number of transmembrane domains, origin, phyletic distribution) of the Tom complex and Tim complexes.

Author’s response: *So far, there has been no similarity found in any aspects of the TIM and TOM complexes and our analyses support that. Tom40 is a beta-barrel protein, which can be found only in the outer membranes of gram-negative bacteria, plastids and mitochondria. Also the receptor subunits and small Toms are exclusive to the TOM complex. The relationship of Tim17 protein to mitochondrial uncoupling protein 2 (and other members of the mitochondrial carrier protein family) rather reflects a similarity among the transmembrane segments of different mitochondrial inner membrane proteins than the true affiliation of the proteins.*

## Additional files

Additional file 1: Table S1. The list of sequences obtained by the Tim17 protein family-specific HHsearch in Uniprot database of reference proteomes. The columns A to E - taxonomic affiliation, the column F- sequence ID, the column F – sequence description, the column H - the affiliation to particular groups of Tim17 protein family (Nogroup indicates that the sequence could not be assigned to any defined subgroups), the column I- the amino acid sequence. (XLS 2637 kb)
Additional file 2: Table S3. The phylogenetic tree of Tim17 family proteins in Newick format. (TXT 301 kb)
Additional file 3: Figure S1. Protein sequence alignment of Tim22 from *Homo sapiens*, *Saccharomyces cerevisiae* and *Neurospora crassa* and HP20 and HP30 from *Arabidopsis thaliana* as performed by MAFFT [38]. The identical and similar residues were highlighted by turquoise and green color, respectively. The threshold value for shading was set to 50 %. (PDF 41 kb)
Additional file 4: Table S2. The overall distribution of Tim17 family protein in eukaryotes. (XLS 135 kb)
Additional file 5: Figure S2. Protein sequence alignment of Romo1/Mgr2, Tim17 and Tim23 from *Saccharomyces cerevisiae*, *Allomyces macrogynus*, *Homo sapiens*, *Monodelphis domestica* and *Trichoplax adherens* as performed by MAFFT [38]*.* The grey cylinders denote the transmembrane regions. The identical and similar residues were highlighted by turquoise and green color, respectively. The threshold value for shading was set to 50 %. (PDF 25 kb)
Additional file 6: Table S4. Prokaryotic proteins carrying a PRAT motif [(G/A)X_2_(F/Y)X_10_RX_3_DX6(G/A/S)GX_3_G]. (PDF 133 kb)

## Abbreviations

HMM: Hidden Markov model
LECA: Last eukaryotic common ancestor
PRAT: Preprotein and amino acid transporter
SAR: Stramenopiles, Alveolata, Rhizaria
TIM23 complex: Translocase of the inner mitochondrial membrane 23 complex
