# Supplementary material for: Evolution of the Tim17 protein family
Source: Biol Direct. 2016 Oct 19;11:54. doi: 10.1186/s13062-016-0157-y (PMC5072337; doi:10.1186/s13062-016-0157-y)
Supplement: Additional file 3: Figure S1. — Protein sequence alignment of Tim22 from Homo sapiens, Saccharomyces cerevisiae and Neurospora crassa and HP20 and HP30 from Arabidopsis thaliana as performed by MAFFT [38]. The identical and similar residues were highlighted by turquoise and green color, respectively. The threshold value for shading was set to 50 %. (PDF 41 kb) [file 13062_2016_157_MOESM3_ESM.pdf]

```
                                   10         20         30         40         50         60         70         80
                           ....|....| ....|....| ....|....| ....|....| ....|....| ....|....| ....|....| ....|....|
Tim22Homo              1   MAAAAP---- ---------- -----NAGGS APETAGSAEA PLQYSLLLQY LVGDKRQPRL LEPGSLGGIP --SPAKSEEQ  59
Tim22Saccharomyces     1   MVYTGFG--- ---------- -LEQISPAQK KPYNELT--- ---------- ---------- ---------P --EEQGERGA  32
TIM22Neurospora        1   MNFPGM---- ---------- ------PGGA APSGGAA--- ---------- ---------- ---------P GGYDPNDPNI  28
HP20_At4g26670         1   MAANDSSNAI DIDGNLDSDS NLN--TDGDE ATDNDSS--- ---------- ---------- ---KALVTIP ----------  42
HP30_2_At5g24650       1   MGKDG----- ---------- ------EGDK KRETMAVMS- ---------- LMKDQQN--- ---------P --IQQFQVKF  34

                                   90        100        110        120        130        140        150        160
                           ....|....| ....|....| ....|....| ....|....| ....|....| ....|....| ....|....| ....|....|
Tim22Homo             60   KMIEKAMESC AFKAALACVG GFV--LGGAF GVFTAGIDTN V--------- ---------- ---GFDPK-- DPYRTPTAKE  113
Tim22Saccharomyces    33   EMIMNFMTSC PGKSVVSGVT GFA--LGGVL GLFMASMAYD TPLHTPTPAN TAATATAGNI GVGGISRTVQ QISDLPFRQQ  110
TIM22Neurospora       29   KMMQKAMESC FAKTVMSGGA GFA--LGGVF GMFMASMAYD TPYHSSTTPG TGPGANPAAA GIPGYKPV-- DLSSMPLKEQ  104
HP20_At4g26670        42   -----APAVC LFRFAGDAAG GAV--MGSIF G-YGSGLFKK K--------- ---------- ---GFKGS-- ----------  80
HP30_2_At5g24650      35   KEIETGFKSW LSKQKLPVEA AVVTAMGGVQ GAFIGGLM-- ---------G TLSPEMPQA- ---GIDPQ-- AMASIKQTQA  97

                                  170        180        190        200        210        220        230        240
                           ....|....| ....|....| ....|....| ....|....| ....|....| ....|....| ....|....| ....|....|
Tim22Homo            114   V---LKDMGQ RGMSYAKNFA IVGAMFSCTE CLIESYRGTS DWKNSVISGC ITGGAIG--- --FRAGLKAG AIGCGGFAAF  185
Tim22Saccharomyces   111   MKLQFTDMGK KSYSSAKNFG YIGMIYAGVE CVIESLRAKN DIYNGVTAGF FTGAGLA--- --YKAGPQAA LMGGAGFAAF  185
TIM22Neurospora      105   LKHGFKDMGQ RSYSTAKNFA KVGALFSGIE CGIEGLRAKN DLGNGVAAGC LTGAILA--- --KNGGPQAA AVGCAGFAAF  179
HP20_At4g26670        80   ----FADAGQ S----AKTFA VLSGVHSLVV CLLKQIRGKD DAINVGVAGC CTGLALS--- --FPGAPQAL LQSCLTFGAF  147
HP30_2_At5g24650      98   L------VGG P-LVQARNFA AITGVNAGIA CVMKRIRGKE DLESAVVAAF GSGVAYSLVS AGLQGQPMNA ITTAAGFAVF  170

                                  250        260        270        280        290        300        310        320
                           ....|....| ....|....| ....|....| ....|....| ....|....| ....|....| ....|....| ....|....|
Tim22Homo            186   SAAIDYYLR- ---------- ---------- ---------- ---------- ---------- ---------- ----------  194
Tim22Saccharomyces   186   SAAIDLYMKS E------DGR PPQNDF---- ---------- ---------- ---------- ---------- ----------  205
TIM22Neurospora      180   SAAIDAWMR- ---------M PSEED----- ---------- ---------- ---------- ---------- ----------  194
HP20_At4g26670       148   SFILEGLNKR QTALAHSVSL RHQTGLFQDH HRALPLSLAL PIPEEIKGAF SSFCKSL--- ---------- -------AKP  207
HP30_2_At5g24650     171   QGVFFKLGER -------FSK PSVEDPYYTR GRSMLLKLGL EKYEK----- -NFKKGLLAD PTLPLLTDSA LRDVSIPPGP  237

                                  330        340
                           ....|....| ....|....| ..
Tim22Homo            194   ---------- ---------- -- 194
Tim22Saccharomyces   205   ---------- ---------- KE 207
TIM22Neurospora      194   ---------- ---------- -- 194
HP20_At4g26670       208   R--------- ---------- KF 210
HP30_2_At5g24650     238   RLLILDHIQR DPELKGKRGS RG 259
```

Supplementary Figure 1
