# Supplementary material for: Evolution of the Tim17 protein family
Source: Biol Direct. 2016 Oct 19;11:54. doi: 10.1186/s13062-016-0157-y (PMC5072337; doi:10.1186/s13062-016-0157-y)
Supplement: Additional file 5: Figure S2. — Protein sequence alignment of Romo1/Mgr2, Tim17 and Tim23 from Saccharomyces cerevisiae, Allomyces macrogynus, Homo sapiens, Monodelphis domestica and Trichoplax adherens as performed by MAFFT [38]. The grey cylinders denote the transmembrane regions. The identical and similar residues were highlighted by turquoise and green color, respectively. The threshold value for shading was set to 50 %. (PDF 25 kb) [file 13062_2016_157_MOESM5_ESM.pdf]

```
Romo1/Mgr2 S. cerevisiae   MP----------------------------------------------------------PLPQNYAQQQPSNWDKFKM------------------------------- 21
Romo1/Mgr2 H. sapiens      MP----------------------------------------------------------VAVGPYGQSQPSCFDRVKM------------------------------- 21
Romo1/Mgr2 M. domestica    MP----------------------------------------------------------VAVGPYGQSQPSCFDRVKM------------------------------- 21
Romo1/Mgr2 T. adherens     M----------------------------------------------------------------PQHYGQPSCFDKVKF------------------------------- 16
Romo1/Mgr2 A. macrogynus  MS-----------------------------------------------------------MMPPPQQQGPSFTDKLKM------------------------------- 20
Tim17 S. cerevisiae        MS----------------------------------------------------------------ADHSRDPCPI------------------------------------ 12
Tim17 H. sapiens           M-----------------------------------------------------------------EEYAREPCPW------------------------------------ 11
Tim17 M. domestica         M-----------------------------------------------------------------EEYAREPCPW------------------------------------ 11
Tim17 T. adherens          M-----------------------------------------------------------------EEYAREPCPY------------------------------------ 11
Tim17 A. macrogynus        MP----------------------------------------------------------------HHDRMRDPCPW------------------------------------ 13
Tim23 M. domestica         ME---GNGGNSNRSSGGLAG---------------------------------FFGGSGGGPSYSNADLAGVPLTGMSPL-----SPYLNVDPRYLIQDTDEFILPTGAN 69
Tim23 H. sapiens           ME---GGGGSGNKTTGGLAG---------------------------------FFGAGGAG--YSHADLAGVPLTGMNPL-----SPYLNVDPRYLVQDTDEFILPTGAN 67
Tim23 T. adherens          MSDDYG--------------------------------------------------KDGYSRDYSSTDPTMSASLSSTNKALLSPYMNIDPRSLHQDGSEFIFAGEPV 58
Tim23 S. cerevisiae        MSWLFGDKTPTDDANAAVGGQDTTKPKELSLKQSLGFE-----------PNINNIISGPGGMHVDTARLHP--LAGLDKG-----VEYLDLEEEQLSSLEG----SQGLI 88
Tim23 A. macrogynus        MS-FFRSFTSSSSSSPDGAPADTSAPVAPAPAPALSQQQQPQQPQQPQPPAIETVGSMLSGLDFTQASRALSPVGQLGSG-----VEYIFTDDSPLHAASG------GFV 98

Romo1/Mgr2 S. cerevisiae   -------GLMMGTTVGVCTGILFGGFAIATQG-----------PGPDGVVRTLGKYIAGSAGTFG----LFMSIGSIIRSDSESSPMSHPNLNLQQQ-ARLEMWKLRAKY 108
Romo1/Mgr2 H. sapiens      -------GFVMGCAVGMAAGALFGTFSCLRIG-----------MRGRELMGGIGKTMMQSGGTFG----TFMAIGMGIRC------------------------------- 79
Romo1/Mgr2 M. domestica    -------GFVMGCAVGMAAGALFGTFSCLRIG-----------MRGRELMGGIGKTMMQSGGTFG----TFMAIGMGIRC------------------------------- 79
Romo1/Mgr2 T. adherens     -------GVMIGFAVGMSSGALFGTYSAFRMG-----------LRGRELLSTVGKIMLQGGGTFG----VFMGIGSAIRC------------------------------- 74
Romo1/Mgr2 A. macrogynus  -------GAMMGCGVGVGLGFVIGNIQYLIYG-----------ARGKGYLGTLANAITTSGASFG----FFMAVGSVIRTEGAREIAFPPHARVTARPAALVSAELRESH 108
Tim17 S. cerevisiae        -------VILNDFGGAFAMGAIGGVVWHGIKGFRNSPLG----ERGSGAMSAIKARAPVLGGNFGVWGGLFSTFDCAVKAVRKREDP---WNAIIA--GFFTGGALAVRG 106
Tim17 H. sapiens           -------RIVDDCGGAFTMGTIGGGIFQAIKGFRNSPVG--VNHRLRGSLTAIKTRAPQLGGSFAVWGGLFSMIDCSMVQVRGKEDP---WNSITS--GALTGAILAARN 107
Tim17 M. domestica         -------RIVDDCGGAFTMGVIGGGVFQAIKGFRNAPVG--IRHRFRGSISAVRIRAPQIGGSFAVWGGLFSTIDCGLVRLRGKEDP---WNSITS--GALTGAVLAARS 107
Tim17 T. adherens          -------RIIDDCGGAFSMGCIGGGVFSLVKGYRNSPPG----KRLVGSITAIKTRAPVLGGNFAVWGGLFSTFDCCLIKLRNKEDP---WNSIGS--GALTGAVLAARG 105
Tim17 A. macrogynus        -------NVLNDAGGAFAMGAIGGGMWHFVKGARNAPRG----ERFPGAISAMKARAPVLGGNFAVWGTLFSTFDCAFAGIRQKEDA---WNSIMS--GTLTGGVLALRS 107
Tim23 M. domestica         KTRGRFELAFFTIGGCCITGAAFGAMNGLRLGLKETQKMSWSKPRNVQILNMVTRQGALWANTLGSLALLYSAFGVVIEKTRGAEDD---INTVAA--GTMTGMLYKCTG 174
Tim23 H. sapiens           KTRGRFELAFFTIGGCCMTGAAFGAMNGLRLGLKETQNMAWSKPRNVQILNMVTRQGALWANTLGSLALLYSAFGVIIEKTRGAEDD---LNTVAA--GTMTGMLYKCTG 172
Tim23 T. adherens          KRRSWGERMFSNVGSSYMIGITCGGAWGLFEGLR-TPHGNTMKLRINGILNSCTRRGPFVGNSLG----CIETILGKVTQKEDEDNP---YNTVGA--AVLTGAIFKSTG 158
Tim23 S. cerevisiae        PSRGWTDDLCYGTGAVYLLGLGIGGFSGMMQGLQNIPPNSPGKLQLNTVLNHITKRGPFLGNNAGILALSYNIINSTIDALRGKHDT---AGSIGA--GALTGALFKSSK 193
Tim23 A. macrogynus        PSRSWTDDLCYGTGTAYLSGLLFGGSWGFVQGMRIPLPVASAKLRVNAVLNAMTRRGPFVANSIGVLALMYNSMNSAITSQVGTANA--PAASVMA--AASAGALFKATS 204

Romo1/Mgr2 S. cerevisiae   GIR-------------------------------------------------------------KD------------------ 113
Romo1/Mgr2 H. sapiens      ------------------------------------------------------------------------------------ 79
Romo1/Mgr2 M. domestica    ------------------------------------------------------------------------------------ 79
Romo1/Mgr2 T. adherens     ------------------------------------------------------------------------------------ 74
Romo1/Mgr2 A. macrogynus  ARMAAMRSSS-------------------------------------------------------------------------- 118
Tim17 S. cerevisiae        GWRHTRNSSITCACLLGVIEGVGLMFQRYAAWQAKPMAPPL--PEAPSSQPLQA------------------------------ 158
Tim17 H. sapiens           GPVAMVGSAAMGGILLALIEGAGILLTRFASAQFPNGPQFAEDPSQLPSTQLPSSPFG---------------------DYRQYQ 171
Tim17 M. domestica         GPLAMVGSAMMGGILLALIEGVGILLTRYTAQQFRNTPPFIEDPGQLPPKEGSAPPPG-----------------YP--GYGQYQ 173
Tim17 T. adherens          GLSSSLRSAAVGGILLALIEGVGIAITRMTAEQFKPGWTTVS------------------------------------------ 147
Tim17 A. macrogynus        GWKAALISATVGGVLLGMIEGAGVVMNRMNADAFRPQAPLL--PPELQQQAGMQPSA--------------------------- 162
Tim23 M. domestica         GLRGVARGGLTGLTLTSL--------YALYNN-WEHMKGSMTQQSL-------------------------------------- 211
Tim23 H. sapiens           GLRGIARGGLTGLTLTSL--------YALYNN-WEHMKGSLLQQSL-------------------------------------- 209
Tim23 T. adherens          GIRATALAAAIGGTLAVT--------YHFGQMIWQKEKPTFSTPNWSS------------------------------------ 198
Tim23 S. cerevisiae        GLKPMGYSSAMVAAACAV---------------WCSVKKRLLEK---------------------------------------- 222
Tim23 A. macrogynus        TLRLDSPSLLFLRSETDP--------TPFSTR-WRPANGHLERHVRVGRSPLASDQANPRGLAICRQDCHALVRLDAPGRGLLVFE 281
```

Supplementary Figure 2
