# Supplementary material for: Evolution of the Tim17 protein family
Source: Biol Direct. 2016 Oct 19;11:54. doi: 10.1186/s13062-016-0157-y (PMC5072337; doi:10.1186/s13062-016-0157-y)
Supplement: Additional file 6: Table S4. — Prokaryotic proteins carrying a PRAT motif [(G/A)X2(F/Y)X10RX3DX6(G/A/S)GX3G]. (PDF 133 kb) [file 13062_2016_157_MOESM6_ESM.pdf]

Supplementary table 3. PRAT motif in prokaryotic proteins

No.	Entry Name	Position	Description
1	ebi:EbC_44000	242..272	bscC; cellulose synthase operon protein C
2	xcc:XCC2930	626..656	yoaA; ATP-dependent helicase
3	xcb:XC_1178	626..656	ATP-dependent helicase
4	xca:xcc-b100_1222	626..656	putative ATP-dependent DNA helicase
5	xtn:FD63_04990	623..653	helicase
6	sml:Smlt0528	709..739	putative NHL repeat protein
7	smt:Smal_0408	135..165	SMP-30/Gluconolaconase/LRE domain protein
8	buj:BurJV3_0430	135..165	NHL repeat containing protein
9	buj:BurJV3_0875	182..212	OmpA/MotB domain protein
10	smz:SMD_0447	94..124	NHL repeat protein
11	smz:SMD_0961	182..212	OmpA domain protein
12	psd:DSC_05750	162..192	thiamine biosynthesis protein
13	psd:DSC_13310	295..325	hemE; uroporphyrinogen decarboxylase
14	psk:U771_21305	212..242	ABC transporter ATP-binding protein
15	slo:Shew_2313	121..151	Fe-S protein assembly chaperone HscA
16	pseo:OM33_08005	368..398	cytochrome C biogenesis protein
17	maq:Maqu_3023	88..118	chaperone DnaJ domain protein
18	mhc:MARHY2962	88..118	cbpA; curved DNA-binding protein, analogue of the DnaJ, co-chaperone of DnaK (Hsp40 family)
19	mad:HP15_2909	93..123	heat shock protein DnaJ domain protein
20	msr:AU15_20210	28..58	hypothetical protein
21	mari:ACP86_04740	88..118	cytochrome C biogenesis protein
22	tvi:Thivi_3349	612..642	DNA helicase, Rad3
23	mpur:MARPU_07805	106..136	methyl-accepting chemotaxis protein
24	aeh:Mlg_1232	671..701	putative membrane protein; K07288 uncharacterized membrane protein
25	mpc:Mar181_2354	346..376	porin gram-negative type
26	but:X994_6345	501..531	phage integrase family protein
27	bph:Bphy_2047	247..277	protein of unknown function DUF477
28	bpy:Bphyt_4736	176..206	hypothetical protein
29	bue:BRPE67_ACDS01190	56..86	uncharacterized protein
30	bue:BRPE67_CCDS11140	249..279	uncharacterized protein precursor
31	axy:AXYL_03112	137..167	Tat pathway signal sequence domain protein 15
32	axo:NH44784_041941	241..271	TonB-dependent receptor; Outer membrane receptor for ferrienterochelin and colicins
33	axo:NH44784_053511	29..59	hypothetical protein
34	axn:AX27061_2371	241..271	TonB-dependent receptor
35	axn:AX27061_3580	29..59	hypothetical protein
36	axs:LH59_16440	29..59	hypothetical protein
37	dac:Daci_4352	494..524	multi-sensor hybrid histidine kinase (EC:2.7.13.3)
38	vap:Vapar_4522	513..543	Polypeptide-transport-associated domain protein ShlB-type
39	vap:Vapar_6356	494..524	Polypeptide-transport-associated domain protein ShlB-type
40	vpe:Varpa_2158	512..542	polypeptide-transport-associated domain-containing protein shlb-type

41	vpe:Varpa_3931	502..532	polypeptide-transport-associated domain-containing protein shlb-type
42	vpd:VAPA_2c07470	520..550	shlB1; putative hemolysin transporter protein ShlB
43	vpd:VAPA_2c09540	509..539	shlB2; putative hemolysin transporter protein ShlB
44	mpt:Mpe_A1216	148..178	thiamine biosynthesis lipoprotein
45	mms:mma_3616	176..206	apbE; thiamine biosynthesis lipoprotein
46	rge:RGE_07540	179..209	hypothetical protein
47	nis:NIS_1752	66..96	hypothetical protein
48	gur:Gura_2011	143..173	beta-hydroxyacyl-(acyl-carrier-protein) dehydratase, FabA/FabZ
49	gbm:Gbem_1975	184..214	modC-1; molybdate ABC transporter, ATP-binding protein
50	gbm:Gbem_2437	302..332	cell division protein, SpoIID family, putative
51	geo:Geob_3148	143..173	polyketide synthase
52	geb:GM18_2234	184..214	molybdate ABC transporter, ATPase subunit
53	ppd:Ppro_2603	26..56	uncharacterized domain 1
54	dpi:BN4_11679	80..110	hypothetical protein
55	dgg:DGI_1393	450..480	putative multi-sensor hybrid histidine kinase
56	ade:Adeh_0117	34..64	SSU ribosomal protein S18P
57	acp:A2cp1_0135	32..62	ribosomal protein S18
58	ank:AnaeK_0124	34..64	rpsR; 30S ribosomal protein S18
59	ank:AnaeK_1325	1212..1242	hypothetical protein
60	mym:A176_005302	164..194	Outer membrane vitamin B12 receptor BtuB
61	vin:AKJ08_2310	73..103	4-diphosphocytidyl-2-C-methyl-D-erythritol kinase
62	scl:sce9355	1768..1798	cellulose synthase 1 operon protein C
63	scu:SCE1572_23355	125..155	hypothetical protein
64	scu:SCE1572_43460	288..318	hypothetical protein
65	hoh:Hoch_6735	79..109	hypothetical protein
66	hoe:IMCC20628_03022	479..509	pseudouridine synthase family protein (EC:5.4.99.22)
67	rec:RHECIAT_CH0000375	441..471	putative carboxypeptidase-related protein (EC:3.4.16.-)
68	rle:RL0352	441..471	putative exported peptidase
69	rlt:Rleg2_4351	442..472	peptidase S10 serine carboxypeptidase
70	rlg:Rleg_4619	443..473	peptidase S10 serine carboxypeptidase
71	rhl:LPU83_pLPU83d0946	165..195	putative secreted protein
72	mor:MOC_1747	70..100	Catalase (EC:1.11.1.6)
73	rva:Rvan_1086	12..42	hypothetical protein
74	rva:Rvan_2815	12..42	hypothetical protein
75	rli:RLO149_c031200	2437..2467	hypothetical protein
76	malg:MALG_01407	171..201	Precorrin-6x reductase (EC:1.3.1.54)
77	mmr:Mmar10_0227	108..138	PfkB domain protein
78	sphm:G432_09150	141..171	hopanoid biosynthesis associated RND transporter-like protein HpnN
79	ssan:NX02_17690	325..355	hypothetical protein
80	rpm:RSPPHO_03231	3..33	Chemotaxis sensory transducer
81	baco:OXB_0786	582..612	hypothetical protein
82	paeh:H70357_32940	257..287	translation factor
83	lpl:lp_3363	378..408	copB; copper transporting ATPase
84	lpj:JDM1_2696	378..408	copB; copper transporting ATPase
85	lpt:zj316_0024	378..408	copB; Copper transporting ATPase (EC:3.6.3.4)
86	lps:LPST_C2765	378..408	copB; copper transporting ATPase
87	lpr:LBP_cg2691	378..408	copB; Copper transporting ATPase
88	lpz:Lp16_2647	378..408	copper transporting ATPase
89	lpb:SH83_13980	378..408	ATPase
90	sth:STH3159	134..164	conserved domain protein

91	mas:Mahau_2082	71..101	4-diphosphocytidyl-2-C-methyl-D-erythritol kinase
92	mtu:Rv3313c	221..251	add; adenosine deaminase
93	mtv:RVBD_3313c	221..251	adenosine deaminase Add
94	mra:MRA_3355	221..251	add; adenosine deaminase (EC:3.5.4.4)
95	mtf:TBFG_13344	221..251	adenosine deaminase add (adenosine aminohydrolase)
96	mtb:TBMG_03362	221..251	adenosine deaminase
97	mtk:TBSG_03385	221..251	adenosine deaminase add
98	mtz:TBXG_003342	221..251	adenosine deaminase add
99	mtg:MRGA327_20415	221..251	adenosine deaminase (EC:3.5.4.4)
100	mti:MRGA423_20825	221..251	adenosine deaminase (EC:3.5.4.4)
101	mte:CCDC5079_3062	221..251	adenosine deaminase add
102	mtur:CFBS_3506	221..251	add; adenosine deaminase
103	mtl:CCDC5180_3023	221..251	adenosine deaminase add
104	mto:MTCTRI2_3381	221..251	add; adenosine deaminase
105	mtd:UDA_3313c	221..251	add; hypothetical protein
106	mtn:ERDMAN_3631	221..251	add; adenosine deaminase
107	mtj:J112_17805	221..251	adenosine deaminase (EC:3.5.4.4)
108	mtub:MT7199_3356	221..251	putative ADENOSINE DEAMINASE ADD (ADENOSINE AMINOHYDROLASE) (EC:3.5.4.4)
109	mtuc:J113_23150	219..249	adenosine deaminase (EC:3.5.4.4)
110	mtue:J114_17770	221..251	adenosine deaminase (EC:3.5.4.4)
111	mtx:M943_17125	221..251	adenosine deaminase
112	mtul:TBHG_03250	221..251	adenosine deaminase Add
113	mtut:HKBT1_3493	221..251	add; adenosine deaminase
114	mtuu:HKBT2_3500	221..251	add; adenosine deaminase
115	mtq:HKBS1_3503	221..251	add; adenosine deaminase
116	mbo:Mb3342c	221..251	add; adenosine deaminase (EC:3.5.4.4)
117	mbb:BCG_3379c	221..251	add; adenosine deaminase (EC:3.5.4.4)
118	mbt:JTY_3339	221..251	add_1; adenosine deaminase (EC:3.5.4.4)
119	mbt:JTY_3359	221..251	add_2; adenosine deaminase (EC:3.5.4.4)
120	mbt:JTY_3379	221..251	add_3; adenosine deaminase (EC:3.5.4.4)
121	mbm:BCGMEX_3377c	221..251	add; Adenosine deaminase (EC:3.5.4.4)
122	mbk:K60_034450	221..251	adenosine deaminase add
123	mbx:BCGT_3177	221..251	Adenosine deaminase (EC:3.5.4.4)
124	mbz:LH58_18015	221..251	adenosine deaminase
125	maf:MAF_33250	221..251	add; adenosine deaminase (EC:3.5.4.4)
126	mce:MCAN_33391	221..251	add; putative adenosine deaminase ADD (adenosine aminohydrolase)
127	mcq:BN44_70108	221..251	add; Putative adenosine deaminase Add (adenosine aminohydrolase) (EC:3.5.4.4)
128	mcv:BN43_60332	221..251	add; Putative adenosine deaminase Add (adenosine aminohydrolase) (EC:3.5.4.4)
129	mcx:BN42_41374	221..251	add; Putative adenosine deaminase Add (adenosine aminohydrolase) (EC:3.5.4.4)
130	mcz:BN45_60354	221..251	add; Putative adenosine deaminase Add (adenosine aminohydrolase) (EC:3.5.4.4)
131	mle:ML0700	221..251	add; adenosine deaminase (EC:3.5.4.4)
132	mlb:MLBr_00700	221..251	add; adenosine deaminase
133	mul:MUL_4210	123..153	galE1; UDP-glucose 4-epimerase GalE1
134	mgi:Mflv_3124	73..103	conserved hypothetical protein
135	mgi:Mflv_4841	221..251	adenosine deaminase (EC:3.5.4.4)
136	msp:Mspyr1_24410	73..103	hypothetical protein
137	msp:Mspyr1_42510	221..251	adenosine deaminase (EC:3.5.4.4)
138	mab:MAB_3890c	198..228	Probable histidine kinase response regulator
139	mabb:MASS_3902	198..228	two-component histidine kinase DevS
140	mmv:MYCMA_2153	198..228	histidine kinase

141	may:LA62_19745	198..228	histidine kinase
142	mabo:NF82_19480	198..228	histidine kinase
143	mabl:MMASJCM_3907	198..228	putative sensor kinase
144	maz:LA61_19645	198..228	histidine kinase
145	mak:LH56_04740	198..228	histidine kinase
146	mys:NF92_04800	198..228	histidine kinase
147	myc:NF90_04795	198..228	histidine kinase
148	mmi:MMAR_5133	123..153	galE1; UDP-glucose 4-epimerase GalE1
149	mli:MULP_05396	123..153	galE1; UDP-glucose 4-epimerase GalE1 (EC:5.1.3.2)
150	mkn:MKAN_05520	63..93	hypothetical protein
151	mkn:MKAN_20790	221..251	adenosine deaminase
152	mks:LG40_05460	43..73	hypothetical protein
153	mks:LG40_20605	221..251	adenosine deaminase
154	mki:LH54_05465	43..73	hypothetical protein
155	mki:LH54_20720	221..251	adenosine deaminase
156	cjk:jk0046	131..161	putative ThiJ/PfpI family protein
157	car:cauri_0738	131..161	ThiJ/PfpI family protein
158	ckp:ckrop_2062	114..144	putative ThiJ/PfpI family protein
159	cii:CIMIT_03240	40..70	oxidoreductase
160	cii:CIMIT_07450	131..161	thiamine biosynthesis protein ThiJ
161	coa:DR71_1702	133..163	DJ-1/PfpI family protein
162	csx:CSING_04185	131..161	Putative amidotransferase
163	csx:CSING_07665	132..162	tdcG; L-serine dehydratase, iron-sulfur-dependent, single chain form (EC:4.3.1.17)
164	cmv:CMUST_01965	4..34	putative DUF1266 family protein
165	rer:RER_20480	220..250	add; adenosine deaminase (EC:3.5.4.4)
166	rey:O5Y_09845	220..250	adenosine deaminase (EC:3.5.4.4)
167	reb:XU06_10080	220..250	adenosine deaminase
168	gbr:Gbro_4631	96..126	SNARE associated Golgi protein-like protein
169	srt:Srot_0620	69..99	preprotein translocase subunit SecG
170	src:M271_08210	47..77	hypothetical protein
171	lxx:Lxx18130	476..506	two-component system, sensor protein
172	mim:AKG07_07185	127..157	4-diphosphocytidyl-2C-methyl-D-erythritol kinase (EC:2.7.1.148)
173	mio:AOA12_01215	502..532	cytochrome C biogenesis protein
174	iva:Isova_1057	142..172	hypothetical protein
175	ars:ADJ73_06540	23..53	enoyl reductase
176	ppc:HMPREF9154_2947	281..311	hypothetical protein
177	fre:Franean1_7288	551..581	serine/threonine protein kinase
178	fri:FraEuI1c_7136	544..574	serine/threonine protein kinase
179	fal:FRAAL3011	720..750	LuxR family transcriptional regulator
180	bsd:BLASA_3360	43..73	putative Dihydrofolate reductase
181	amd:AMED_4809	157..187	MerR family transcriptional regulator
182	amn:RAM_24480	157..187	MerR family transcriptional regulator
183	amm:AMES_4750	157..187	MerR family transcriptional regulator
184	amz:B737_4750	157..187	MerR family transcriptional regulator
185	kphy:AOZ06_32210	515..545	hypothetical protein
186	afs:AFR_01450	484..514	putative DEAD/DEAH box helicase
187	acq:AM609_06650	132..162	thiamine biosynthesis protein ThiJ
188	rxy:Rxyl_0550	88..118	hypothetical protein
189	gpa:GPA_30220	268..298	hypothetical protein
190	apv:Apar_0409	60..90	transcriptional regulator, LysR family

Supplementary table 3. PRAT motif in prokaryotic proteins

191	taz:TREAZ_2244	617..647	peptidase, U32 family
192	gma:AciX8_3091	94..124	cobalamin 5'-phosphate synthase
193	sus:Acid_1225	495..525	NHL repeat containing protein
194	hao:PCC7418_0289	288..318	photosystem I core protein PsaB
195	gei:GEI7407_1208	986..1016	protein of unknown function DUF490
196	arp:NIES39_N00970	158..188	hypothetical protein
197	pgi:PG1715	759..789	hypothetical protein
198	pgn:PGN_0400	798..828	hypothetical protein
199	pgt:PGTDC60_0586	788..818	hypothetical protein
200	afd:Alfi_1149	243..273	Acyl-protein synthetase, LuxE
201	ash:AL1_22280	243..273	Acyl-protein synthetase, LuxE.
202	doi:FH5T_20350	148..178	HupE / UreJ protein
203	hhy:Halhy_2969	198..228	peptidase S8 and S53 subtilisin kexin sedolisin
204	hmu:Hmuk_1595	352..382	oligopeptide/dipeptide ABC transporter, ATPase subunit
205	htu:Htur_5093	223..253	hypothetical protein
206	hah:Halar_3531	264..294	protein of unknown function DUF87
